# Perspectives of adolescents with severe obesity on social Media in Preparation for weight-loss surgery: a qualitative study

**DOI:** 10.1186/s12887-020-1992-7

**Published:** 2020-03-02

**Authors:** Elizabeth Prout Parks, Darra D. Finnerty, Jennifer Panganiban, Rosemary Frasso, Chanelle Bishop-Gilyard, Colleen M. Tewksbury, Noel N. Williams, Kristoffel R. Dumon, Gaby Cordero, Douglas L. Hill, David B. Sarwer

**Affiliations:** 1 Division of Gastroenterology, Hepatology, and Nutrition, Children’s Hospital of Philadelphia, 34th Street and Civic Center Boulevard, Philadelphia, PA 19104-4399 USA; 2 The Healthy Weight Program, Children’s Hospital of Philadelphia, 34th Street and Civic Center Boulevard, Philadelphia, PA 19104-4399 USA; 3 Perelman School of Medicine at the University of Pennsylvania, 423 Guardian Drive, 1139 Blockley Hall, Philadelphia, PA 19104 USA; 4 Children’s Hospital of Philadelphia, 2716 South Street, Room 14361, Philadelphia, PA 19146 USA; 5 Center for Public Health Initiatives at the University of Pennsylvania, 144 Anatomy Chemistry Building, 3620 Hamilton Walk, Philadelphia, PA 19104 USA; 6 Department of Psychiatry, Perelman School of Medicine, University of Pennsylvania, Philadelphia, PA 19104 USA; 7 Penn Metabolic Bariatric Surgery Program, Department of Surgery, University of Pennsylvania, Philadelphia, PA 19104 USA; 8 Pediatrics Advanced Care Team, Children’s Hospital of Philadelphia, Philadelphia, PA 19104 USA; 9 College of Public Health, Center for Obesity Research and Education, Temple University, 3223 N. Broad St., Suite 175, Philadelphia, PA 19140 USA

**Keywords:** Social media, Adolescent obesity, Bariatric surgery, Support groups

## Abstract

**Background:**

Currently the most effective treatment for severe obesity in adolescents is weight-loss surgery coupled with lifestyle behavior change. In preparation for weight-loss surgery, adolescents are required to make changes to eating and activity habits (lifestyle changes) to promote long term success. Social media support groups, which are popular among adolescents, have the potential to augment preoperative lifestyle changes. The purpose of this study was to qualitatively assess the perceived role of social media as a support tool for weight-loss, and to identify motivators and constraints to lifestyle changes and social media use in adolescents preparing for weight-loss surgery.

**Methods:**

Thematic analysis of social media comments from 13 (3 male, 10 female) adolescents aged 16 ± 1.3 years with a body mass index (BMI) 45 ± 7.3 kg/m^2^ enrolled in a weight-management program preparing for bariatric surgery and who participated in a 12-week pilot social media intervention was performed. Participants commented on moderator posts and videos of nutrition, physical activity, and motivation that were shared three to four times per week. Social media comments were coded using NVivo 11.0 to identify recurrent themes and subthemes.

**Results:**

1) Social media provided accountability, emotional support, and shared behavioral strategies. 2) Motivators for lifestyle changes included family support, personal goals, and non-scale victories. 3) Challenges included negative peers, challenges with planning and tracking, and time constraints.

**Conclusion:**

Adolescents considering bariatric surgery identified social media as a tool for social support and reinforcement of strategies for successful behavior change. Important motivators and challenges to lifestyle changes were identified.

## Background

The prevalence of severe obesity BMI ≥ 120% of the 95th percentile or BMI ≥ 35 kg/m^2^) in adolescents (ages 12 to 21 years) remains elevated at 7.7% and is associated with impairments in quality of life and significant comorbidities such as type 2 diabetes, cardiovascular disease, obstructive sleep apnea [1]. Although studies are limited, bariatric surgery coupled with lifestyle behavior change has documented weight-loss achievements of 30% and significant improvements in glycemic control, cardiovascular disease risk compared with 5% or less with lifestyle interventions alone for the treatment for severe obesity in adults and adolescents [2–6]. To encourage long-term lifestyle behavior change, most third-party payers require medical weight management as a part of the preoperative assessment process for prior authorization. Medical weight management typically consists of 3 to 6 months of preoperative dietary, activity, and lifestyle counseling and is typically completed concurrently with medical, surgical, nutritional, and psychological evaluations [7]. Although the preoperative assessment process is largely viewed as a period to foster engagement between the patient and program as well as to motivate patient adherence, little is known about effective ways to support adolescents in order to achieve these goals.

Previous studies have suggested that maintaining engagement in a weight loss program is associated with larger weight losses [8, 9]. Support group attendance in adolescents is associated with increased clinic attendance which when seen in adults, has been associated with weight-loss and post-operative outcomes [10, 11]. Increases in internet use has led to the use of social networking as a vehicle for the delivery of healthcare information [12]. According to survey data, approximately 95% of adolescents in the United States use social media and have internet access [13]. Preliminary data suggests that capitalizing on adolescent use of social media for peer support may be a method to promote engagement, meet health goals, and potentially enhance patient retention in a medical weight management pre-operative program [9, 14–16]. Preoperative assessment with adolescents preparing for bariatric surgery may be an ideal time period to leverage this tool.

Although bariatric surgery produces significant weight loss in adolescents, additional interventions are necessary for ongoing weight management [17]. To date, there has been limited evidence with regard to the use of social media as a weight loss modality or support platform in adolescents with severe obesity [15, 18–23]. The use of social media can avoid the challenges of travel, missing work and school that hinder in-person interventions. However, for social media to be a successful option for treatment augmentation and adherence, the adolescent perception of social media use, and perceived facilitators and barriers to treatment need to be examined. The objectives of this study were to utilize qualitative methods to: 1) assess the perceived role of social media as a support tool for weight-loss, and 2) identify motivators and constraints to lifestyle changes in adolescents with severe obesity preparing for weight-loss surgery.

## Methods

This is a qualitative analysis utilizing data from a single-arm feasibility pilot study, Facilitating Accomplishments through a Community Experience (FACE) conducted in participants from Southeastern Pennsylvania. The results of the main study have been provided elsewhere [16]. Briefly, males and females aged 13 to 20 years were eligible if their BMI (m/kg^2^) was ≥35, they had a cell phone with a data plan and texting capabilities, and were enrolled in a medical weight management program in preparation for surgery. In the medical weight management program, youth attended monthly in-person clinic visits with 1 or 2 providers including: a dietitian, physician, nurse practitioner, exercise physiologist, or psychologist, for 45 min and a monthly in person support group lead by a clinical psychologist. Participants were excluded if they had syndromic or secondary obesity, a developmental delay, untreated depression or psychosis, an eating disorder other than binge eating disorder, or active substance abuse.

### Intervention

Participants were enrolled into one of two private social media Facebook groups based upon age (14 to 16 and 17 to 20 years). Participants met the members of their group in person once prior to the start of the 12 week intervention, and at the conclusion of the intervention. The intervention included a social media facilitator (an adolescent clinical psychologist with more than 10 years of experience with patients preparing for surgery and obesity treatment), who posted video content from experts in weight management and peers three times per week on the study’s private social media platform. Experts included: dietitians, the medical director of the adolescent bariatrics program (with specialty training in pediatrics, nutrition and obesity medicine), an adolescent psychologist, and exercise physiologists. Topics discussed included self-monitoring of food and exercise, at home and gym fitness demonstrations, eating away from home, label reading, foods related to weight gain, holiday eating, body image and self-esteem, meal replacements, nutrition after surgery, best changes after surgery, side effects of surgery, medications and vitamins, and life before and after surgery. The adolescents “commented” or “liked” moderator and/or peer posts, and also posted their own individual posts.

This intervention was informed by two established theories of behavior change: Bandura’s Social Learning Theory and the Integrative Model of Health Behavior developed by Fishbein and Yzer [24]. Participants observed models of the desired behavior of changes in videos and were then provided opportunities to practice behavior changes with positive reinforcement provided by social media and members of the group. The behavioral targets in this intervention were all factors associated with weight-loss including: self-monitoring, nutrition, physical activity, medication adherence, and behavioral change strategies [25].

Participants received a US $25 monthly stipend for 3 months (12 weeks) to offset the cost of their phone data plan. Youth were informed that in order to receive the stipend, they needed to post, like, or comment in Facebook at least 3 times per week. Beyond those guidelines, participants could participate in the group as much or as little as desired. There was no increase in compensation for the type of participation/posts (likes vs. comments, photos/videos). This study was approved by the Institutional Review Board at Children’s Hospital of Philadelphia (CHOP) and all participants provided written informed consent or assent depending on their age. The results of the quantitative pre-post-test pilot study are described elsewhere [16].

### Thematic analysis of social media comments

Thematic analysis of social media comments was conducted. All available entries were checked for accuracy and stripped of identifying information. All participant comments were analyzed together although participants were in two separate age groups secondary to a small sample size and lack of age-specific comments. The study team then developed a coding dictionary. Codes were developed through line-by-line reading of a subsample of social media entries and were informed by the literature. Each code was given an explicit definition to ensure coding accuracy and to improve intercoder reliability [26]. Two members of the multidisciplinary research team (authors DF and JP) independently coded all data using NVivo 11.0 (QSR International Pty Ltd., Burlington, MA), a software program for qualitative data coding and analysis [27]. Coding was supervised by a qualitative research scientist (RF). Discrepancies in coding were resolved by consensus and reviewed with the principal investigator (EP). After all data were coded, the team (DF, JP, RF, and EP) met to organize the codes into thematic categories.

## Results

Thirteen adolescents with a mean age of 16 ± 1.30 years enrolled and completed the 12 week social media intervention. See Table 1 for participant baseline characteristics. Secondary to rapid insurance approval for surgery, two adolescents had the sleeve gastrectomy earlier than anticipated: one with surgery 2 weeks prior to the baseline visit and one with surgery 4 weeks into the 12-week intervention. Comments from these subjects were included as they did not deviate from the themes of the rest of the group. Monitoring this additional data was important in order to track potential bias of these individuals and provide a richer understanding of the experience.

**Table 1:** Participant Baseline Characteristics (*N* = 13)

Adolescent Characteristics	Mean (SD) or%
Age, years [mean (SD)]	16.0 (1.30)
Age range, years	14–20
Sex, Male, n	4
Weight, kg [mean (SD)]	127.0 (20.00)
BMI,^a^ kg/m^2^ [mean (SD)]	45.5 (7.30)
BMI-z-score [mean (SD)]	2.52 (0.2)
Race, African American, n	6
Medicaid coverage, n	5

^a^*BMI* body mass index

Thematic analysis approach was used to examine the participant generated content on the social media platform [28]. Analysis of intercoder reliability for this study revealed near perfect agreement (mean κ = .99; range, 0.84–1.00). This result was supported by percentage of agreement analysis, which yielded a mean of 99% (range, 93 to 100%) agreement of all codes [26]. Resultant codes were organized into three thematic categories: 1) social media experience, 2) motivators for lifestyle change, and 3) perceived barriers to lifestyle changes and social media participation, with subthemes as needed. Table 2 provides an overview of themes, subthemes, and illustrative quotes.

1. Social media experience: Participants described their social media experience with the following subthemes: *Accountability* (“having accountability really keeps me on track, thanks FACE.”); *Emotional support* (“The Facebook group gave me a lot of mental support and ideas that I didn’t figure I could get.”); and *Shared Tips for Success* (“Trying to incorporate some veggies any way I can. Since getting protein has to be my priority it’s hard to get fibrous foods into meals, but I’ve been focused on eating green veggies and cauliflower more by adding it to my chicken dishes.”).
2. Motivators for lifestyle change: The following subthemes of motivators for lifestyle changes were described: *Family support* (“My family is doing what I’m doing…and giving me an extra push.”); *Personal goals and aspirations* (“My long term goal is to meet the weight requirement so I will be able to enlist in the Air Force*.*”); and *Non-scale victories- (“*I sat with my legs crossed.”). Non-scale victories were secondary changes in health other than weight-loss.
3. Perceived challenges to making lifestyle changes and social media participation: The following subthemes for perceived challenges were

- *Time constraints*-.(*“*Just like you I think I should be posting and commenting more than usual, but between school and work it is a little challenging but I am trying my best.*”)*
- *Negative peers* – (*“*Unfortunately I have a friend whose exact words were ‘I don’t want you to lose too much weight because I don’t want to be the ugly one.*”); Planning and tracking difficulties*- (*“*Tracking is hard for me because I spend most of my day at school. It was embarrassing, pulling out a piece of paper or something to write down what I was eating.*”*); and Equipment failures- (*“I was supposed to ride my stationary bike every day for thirty minutes while studying. It didn’t happen because I found out the chair was broken.”)*

**Table 2:** Overview of Themes and Illustrative Quotes

Themes	Illustrative Quotes
Social Media experiences	
*Accountability*	*“*Good afternoon everyone! I just wanted to write this post to say I love reading/learning from you guys comments, questions and post… Thanks A lot < 3” “It was good to feel accountable. I know that I have to track calories and record my experiences or I won’t get paid for the study So having accountability really keeps me on track. Thanks, FACE”
*Social and emotional support*	“Everyone always says it’s easier to lose weight and be healthy when you have friends or other people doing it with you, so I think this (FACE) is great because of that.” “I think the support of this group has helped a lot with the things that have nothing to do with weight loss!” “The Facebook group gave me a lot of mental support and ideas that I didn’t figure I could get.”
*Shared tips and strategies for success*	
Tips for tracking	“Ok, sometimes I also get confused, how do I measure my school lunch to put it on my fitness pal, so I’ll just take pictures.” “Ever since I got a smartphone it is so much easier because nobody knows what I’m doing. I love it! Plus it makes tracking so much easier.”
Healthy eating strategies	“Trying to incorporate some veggies any way I can. Since getting protein has to be my priority it’s hard to get fibrous foods into meals, but I’ve been focused on eating green veggies and cauliflower more by adding it to my chicken dishes.” “I have a schedule!! I wrote myself a schedule of when to eat and I try my best to stick to it. PLANNING meals and snacks in advance is the key!! Otherwise I get hungry and over eat or eat the wrong thing.”
Shared nutrition Knowledge	“Foods that are related to weight gain are generally processed foods like soda and chips, or other sort of fast food things like burgers and pizza. I think this because the food doesn’t have anything sort of natural; it’s just packed with sugar and fat.” “I get surprised at how much sugar I have (and sometimes am) consuming.”
Motivators for behavior change	
*Family support*	“First thing that pops in my mind is all the unhealthy food we threw away and how my family is doing what I am doing and giving me an extra push. “ “My family likes to take walks around the park, play just dance 4 together, try different vegetables for dinner and eat dinner as a family.”
*Personal goals and aspirations*	“This week I actually have a different type of fitness goal. For this semester at school, I have physical education. I decided I am actually going to take physical education seriously this year and work hard in it. Not only will it help my body, but it will help my GPA if I do a good job!” “My long term goal is to meet the weight requirement, so I will be able to enlist in the Air Force.” “This past summer my doctor told me I was showing signs of pre-diabetes. This was the first time I had really faced any medical back lash because of my weight. I made the commitment to lose a significant amount of weight that day. I don’t want to be unhealthy.”
*Non-scale victories*	“Since I have been exercising and eating lighter meals as well as drinking just water I feel more refreshed, more comfortable, and motivated:)” “Non-scale victories are my favorite kind, today I sat with my legs crossed during class!! Such a small, yet important non-scale victory.” “Walking is a good stress reliever for me and it’s even more relaxing when you got a nice playlist of music to listen to!”
Perceived challenges to social media group participation and healthy behavior change	
*Negative peers*	“Unfortunately I have a friend whose exact words were ‘I don’t want you to lose too much weight because I don’t want to be the ugly one.’ It sucks that there are people like this who will try and call themselves my friends.” “One of the most valuable lessons I’ve learned so far is that people are afraid of change especially when they think that change will cause someone to do better than them. Just because everyone around you doesn’t support you doesn’t mean you still won’t achieve your goals.”
*Challenges with planning and tracking*	“Dining out, I’d like to say that you can dine out every once in a while but you have to be good with eating good and exercising the week before so you can go out and eat peacefully.” “I’d like to be able to say I hit the gym, which I know is a great stress reliever, but honestly I don’t immediately think to turn to exercise. I guess nowadays I try and take a nap if I can or spend time with my boyfriend if I’m feeling stressed.” “Tracking is hard for me because I spend most of my day at school. It was embarrassing, pulling out a piece of paper or something to write down what I was eating.”
*Time constraints*	“Just like you I think I should be posting and commenting more than usual, but between school and work it is a little challenging but I am trying my best.” “Due to personal things such as school, sticking with my diet…and I also broke my phone (no worries I got it replaced now). “ “School and making sure I’m getting enough sleep has been consuming my life for the past couple weeks.”

## Discussion

Adolescents with severe obesity preparing for bariatric surgery reported that a private social media group provided accountability, social support and strategies for healthy behaviors. Reported challenges to social media and behavioral health change participation included time constraints. Additional perceived challenges to making lifestyle changes were negative peers, difficulty with planning and tracking. Motivations for lifestyle changes while preparing for surgery identified were family support, personal goals and aspirations, and non-scale victories.

Adolescents undergoing bariatric surgery require ongoing psychosocial support which can be augmented in a support group [29, 30]. A social media outlet could deliver a platform in which it provides enough privacy but a venue of where these adolescents can find support from other peers without judgment. Similar to in-person weight management groups, the social media group offered the feeling of being part of a close community that allowed patients to draw on the support of their peers [18].

The social media platform permitted adolescents to share their common barriers with severe obesity face including the difficulties of committing to healthier changes. Even with the ability to login to the support group at the convenience of the participant, challenges to participations included other commitments such as school or work, time constraints, lack of support, and breakdown of exercise equipment. This reflects the inherent challenge of time management and setting priorities, requiring higher levels of executive function which have been found to be diminished in adolescents with obesity [31]. Similar time constraints were identified in qualitative study exploring perspectives of adolescents enrolled in weight management programs without social media support [32].

Interventions using social networking have been shown to lead to reduction in BMI in adults with obesity and offer the potential to be much more practical for day-to-day use when compared to traditional approaches that include cost and time for travel [33, 34]. This study coupled with prior work suggests the potential acceptability and feasibility of social media in the preoperative preparation process [18]. Prior work has demonstrated that utilizing a private social media platform can provide successful positive engagement and increased clinic attendance in adolescents with severe obesity [16]. This study illustrates reasons a social media group can contribute to clinical care specifically for adolescents.

This study highlights the importance of family involvement in surgery preparation for adolescents. This result is consistent with prior findings that positive family and peer support is beneficial for weight loss [35–37]. Interventions involving a family component may increase positive health outcomes in adolescents as well as improve parent’s awareness of their child’s health and well-being [35, 38]. Finding ways to include parents in adjunct social media groups may be important to demonstrate ways to be supportive and to foster positive support.

The study had several strengths and some limitations. Despite the small sample size (*n* = 13) of the pilot study, we were able to reveal meaningful qualitative themes. All participants enrolled in the study completed the intervention. This research explored relevant topics of adolescents with severe obesity from Southeastern Pennsylvania. Consequently, findings are not necessarily transferrable to adolescent groups in other settings. Adolescents were not required to comment and did not receive any further increase in incentives/compensation for providing comments as opposed to providing likes. However, because incentives were not based on the content of the comments, the incentives did not introduce bias in the comments. Although social bias was a possibility, adolescents openly included self-incriminating information (i.e. forgetting to track). The comments were analyzed from a private Facebook group, which was the most popular social media platform used in our age group at the time [39]. It is unlikely that the social media platform would influence the comments of the participants, but it was not possible to assess this possibility. Although according to Pew Research Forum 2018, Facebook is no longer the most popular social media platform, studies have shown that adolescents utilize multiple platforms for different purposes (texting, video chatting, gaming, etc.) [13, 40]. Further, Facebook allowed the ability to have a private group and allowed for the continued ability to access posts, which is important for a support group. Given the changing environment of social media, future qualitative and mixed-methods studies are needed to gain insight into their future incorporation into clinical practice. Two adolescents who underwent surgery were included along with others in preparation. The presence of participants having already had surgery could have influenced group participation. However, the topics and comments from participants specifically related to post-operative behaviors were not included in the analysis as the emphasis was on pre-operative lifestyle behavior changes. To our knowledge, this pilot study is the first to qualitatively analyze social media use as a platform to deliver evidence based weight loss support to adolescents with severe obesity preparing for bariatric surgery.

## Conclusion

Thematic analysis of social media comments from adolescents provided insights into perceived benefits and challenges of social media, factors that provided motivation, and barriers to lifestyle changes in preparation for surgery. After surgery, youth will need to continue to engage in healthy lifestyle changes for weight maintenance. Future research should include parents and explore the use of social media to keep adolescents engaged postoperatively. Understanding motivators and challenges for adolescents for lifestyle changes are critical to provide the appropriate supportive strategies for success.

## Data Availability

All data generated and/or analyzed during the current study are available from the corresponding author on reasonable request.

## Abbreviations

BMI: Body Mass index
CHOP: Children’s Hospital of Philadelphia
FACE: Facilitating accomplishments through a community experience
